# Bioactive Antiviral Black Organic Coatings Functionalized with Silver–Silica and Silver–Zirconium for Cytocompatible Biomaterial Surfaces Against Human Coronavirus

**DOI:** 10.3390/ijms27219399

**Published:** 2026-10-22

**Authors:** Maja A. Zaczek-Moczydłowska, Bartosz Kopyciński, Izabella Biesiada, Purusottam Mishra, Krzysztof Matus, Saeid Ghavami, Ewa Langer, Marek J. Łos

**Affiliations:** 1 Biotechnology Centre, The Silesian University of Technology, 44-100 Gliwice, Poland; 2 Doctoral School, The Silesian University of Technology, 44-100 Gliwice, Poland; 3 Łukasiewicz Research Network-Institute of Polymer Materials, 87-100 Torun, Poland; 4 Faculty of Mechanical Engineering and Technology, The Silesian University of Technology, 44-100 Gliwice, Poland; 5 Department of Human Anatomy and Cell Science, Rady Faculty of Health Sciences, Max Rady College of Medicine, University of Manitoba, Winnipeg, MB R3E 0T6, Canada; 6 Faculty of Medicine, Academy of Silesia, 40-555 Katowice, Poland; 7 Paul Albrechtsen Research Institute, Cancer Care Manitoba, Winnipeg, MB R3E 0V9, Canada

**Keywords:** functional coatings, silver-based additives, antiviral activity, HCoV-229E, cytotoxicity

## Abstract

Bioactive antiviral biomaterial coatings capable of rapidly inactivating respiratory viruses while preserving cytocompatibility are highly desirable for development protective and high-contact biomedical materials. Here, we engineered black (Bl) organic coatings functionalized with two silver (Ag)-based inorganic additives—nano (n) Ag supported on colloidal silica (SiO_2_) and Ag-substituted zirconium phosphate—to develop multifunctional antiviral biomaterial surfaces. Their antiviral efficacy was evaluated against human coronavirus (HCoV) 229E, a model enveloped respiratory coronavirus, together with their cytocompatibility toward human keratinocytes (HaCaT) and human lung fibroblasts (MRC-5) cells. The morphology and elemental composition of the coatings were characterized using scanning electron microscopy (SEM) coupled with energy-dispersive X-ray spectroscopy (EDS). Antiviral performance was quantified by a neutral red uptake cytopathic (NRU-CPE) infectivity assay following 30 and 60 min of virus–surface contact. Coatings functionalized with nAg supported on SiO_2_ reduced infectious virus recovery by 2.2–5.3 log_10_, whereas coatings containing Ag-substituted zirconium phosphate achieved reductions of 1.9–4.7 log_10_, depending on additive loading and exposure time. Interestingly, the unmodified black organic coating also reduced viral infectivity, indicating that both the coating matrix and Ag-based functionalization contribute to the overall antiviral performance. Importantly, all functionalized coatings maintained cell viability above the ISO 10993-5 acceptance threshold (≥70%), demonstrating excellent cytocompatibility.

## 1. Introduction

HCoVs can remain viable on environmental surfaces for extended durations, rendering surface persistence a significant factor in the indirect transmission of respiratory viral infections, especially within healthcare environments and other areas with high contact rates [1,2]. While respiratory droplets and aerosols are recognised as the primary pathways for coronavirus transmission, contaminated surfaces (fomites) may also facilitate viral dissemination under specific circumstances [3]. The longevity of HCoVs on surfaces is influenced by both the viral strain and the type of material. For instance, HCoV-OC43 and various severe acute respiratory syndrome coronavirus 2 (SARS-CoV-2) variants have been documented to retain their infectiousness for as long as 48–72 h on surfaces such as stainless steel, plastics, and touchscreen glass, whereas SARS-CoV-2 has also demonstrated effective transfer from contaminated non-porous surfaces to human skin [4,5]. These findings underscore the potential significance of contaminated surfaces in maintaining viral infectivity and highlight the necessity for strategies aimed at minimizing viral persistence.

In addressing this challenge, significant resources have been allocated to the creation of functional biomaterial coatings that possess inherent antiviral activity [6,7,8]. Of the various strategies available, antiviral coatings stand out as especially appealing due to their ability to offer ongoing, passive protection without the need for frequent chemical disinfection [9,10,11,12,13]. These coatings hold great potential for use on commonly handled polymeric materials, such as personal protective equipment, textiles, and other surfaces that are exposed to high levels of contact.

Ag-based nanomaterials have garnered significant interest due to their extensive antiviral properties against enveloped viruses, including HCoVs and SARS-CoV-2 [14]. Prior research has suggested various mechanisms that contribute to this antiviral effect. Silver nanoparticles (AgNPs) and the Ag^+^ ions they release may interact with viral envelopes and surface glycoproteins, such as spike proteins, which diminishes the ability of viruses to attach and enter host cells [15,16]. Moreover, AgNPs have been shown to enhance the production of reactive oxygen species (ROS), resulting in oxidative damage to viral proteins, lipids, and nucleic acids. Additionally, Ag species may disrupt viral RNA and intracellular replication processes, thereby decreasing viral infectivity [10,17]. While these mechanisms have been discussed in earlier studies, the current research focuses on assessing the functional antiviral efficacy of the coatings rather than directly quantifying Ag^+^ release, ROS production, viral binding, envelope disruption, or other specific molecular mechanisms of viral inactivation. Furthermore, the immobilisation of Ag within inorganic supports such as silica (SiO_2_) or zirconium phosphate matrices can enhance the stability of NPs, control Ag^+^ release, and enable their integration into coating systems, all while sustaining prolonged antiviral activity [9,18].

Despite the increasing interest in antiviral coatings, there are several studies that have systematically compared the intrinsic antiviral properties of the coating matrix itself with those conferred by Ag-supported additives, while also assessing cytocompatibility using both skin- and lung-relevant cell models. Such comparative analyses are crucial for ascertaining whether Ag-containing additives offer significant biological advantages beyond those provided by the coating matrix alone. Consequently, the objective of this study was to evaluate and compare the antiviral efficacy and cytocompatibility of Bl organic coatings, both with and without Ag-based loading additives. In particular, coatings that incorporate AgNPs supported on colloidal SiO_2_ (Ag-SiO_2_) and Ag-substituted zirconium phosphate were compared to their corresponding baseline organic coating to ascertain whether the Ag-containing additives impart additional antiviral activity. The antiviral efficacy was measured against HCoV-229E, a commonly accepted model for enveloped HCoVs, employing a cytopathic effect (CPE) assay to quantify the infectious virus recovered from coated surfaces after specified exposure durations. Therefore, the results should be interpreted as indicative of surface antiviral activity against a representative HCoV model, rather than as direct evidence of efficacy against SARS-CoV-2. Cytocompatibility was assessed using human keratinocyte HaCaT cells and human lung fibroblast MRC-5 cells to evaluate potential cytotoxicity relevant to skin and respiratory exposure. By integrating antiviral efficacy with cytocompatibility evaluation, this study offers a thorough comparison of Ag-functionalized Bl organic coatings for prospective applications on polymer-based surfaces, including protective masks and textiles.

## 2. Results

### 2.1. Characteristics of Bl Coatings

**Figure 1:**
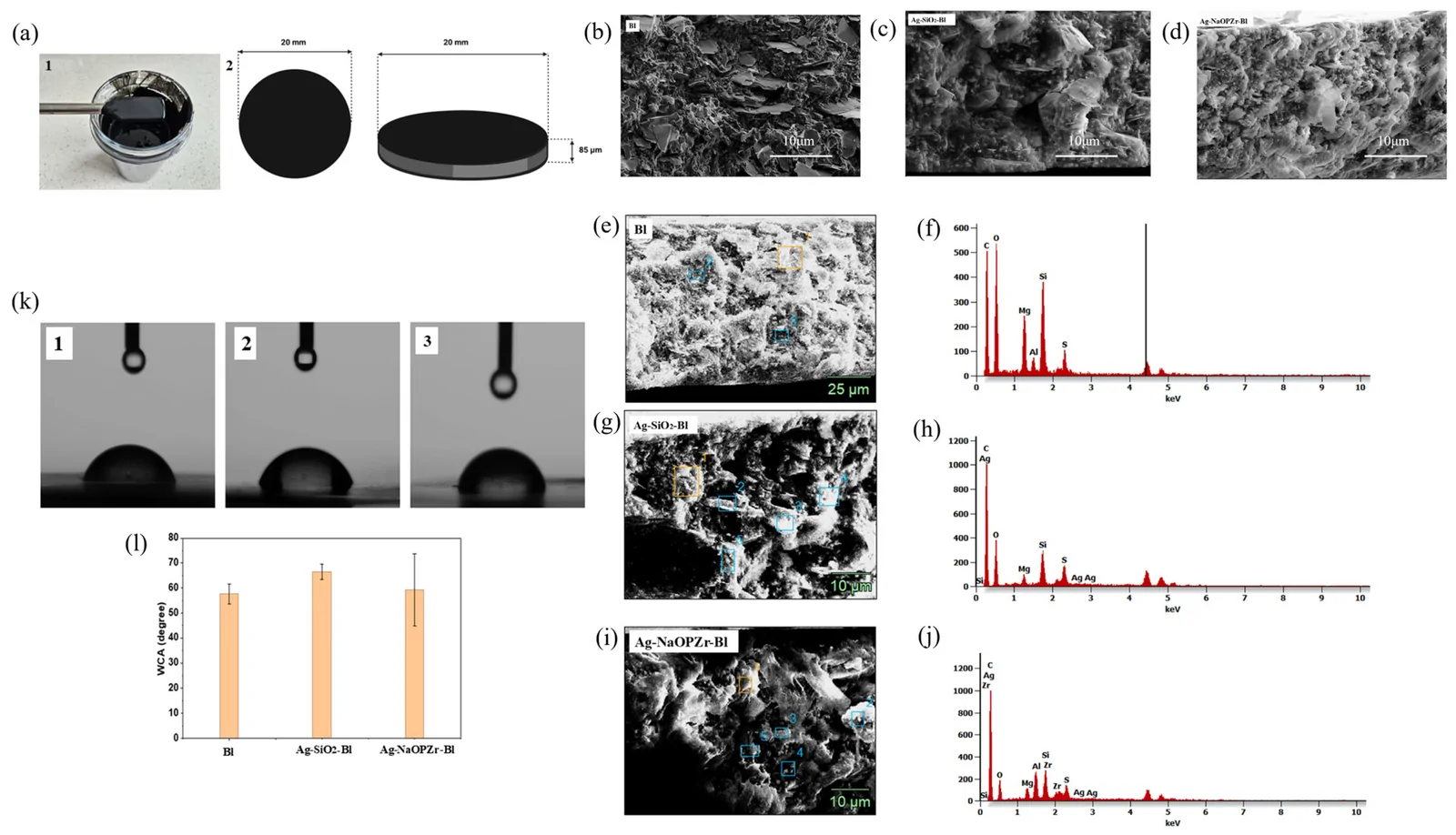
Selected characteristics of Bl coatings. (**a**) Bl coatings; (**1**) Appearance of Bl coating formulation; (**2**) Schematic illustration of the Bl coating disc with the coating geometry and dimensions. SEM micrographs of the coating surface. (**b**) Bl; (**c**) Ag-SiO_2_-Bl, and (**d**) Ag-NaOPZr-Bl. Cross-sectional SEM images with selected EDS analysis points. (**e**) Bl; (**g**) Ag-SiO_2_-Bl, and (**i**) Ag-NaOPZr-Bl. Corresponding EDS spectra are shown in (**f**) Bl; (**h**) Ag-SiO_2_-Bl; (**j**) Ag-NaOPZr-Bl, confirming the elemental composition of each Bl coating. (**k**) Water contact angle measurements of Bl coatings: (**1**) Bl; (**2**) Ag-SiO_2_-Bl; (**3**) Ag-NaOPZr-Bl. (**l**) Graph presenting WCA measured for Bl, Ag-SiO_2_-Bl, and Ag-NaOPZr-Bl coatings.

Bl coating formulations (Figure 1(a1)) were subsequently prepared for antiviral and cytotoxicity testing in the form of discs (Ø 20 mm, ~85 µm thick) (Figure 1(a2)).

The incorporation of Ag–SiO_2_ and Ag–NaOPZr additives had only a minor effect on the density (1.22–1.23 g/cm^3^) and non-volatile content (49–50%) of the coatings (Table 1). The Ag–SiO_2_ formulations exhibited higher viscosity than the reference coating, with the 5 wt.% sample showing the highest value, while the Ag–NaOPZr-modified coatings displayed viscosities comparable to the Bl formulation (Table 1). All coatings retained a low-gloss, matte appearance, although the Ag–NaOPZr-Bl showed noticeably higher gloss at an 85° measurement angle (Table 1). Pendulum hardness ranged from 153 to 172 s, corresponding to 35–40% of the glass standard (430 s), indicating that the incorporation of the additives had only a limited influence on the mechanical hardness of the coatings (Table 1).

SEM images illustrate notable variations in surface structure among the Bl coatings. The Bl coating displays a markedly heterogeneous and rough surface, featuring irregular agglomerates and layered characteristics that are typical of polymer–mineral composite matrices (Figure 1b). In contrast, the Ag-SiO_2_-Bl coating presents a more advanced microstructure with densely packed irregular domains (Figure 1c), indicating effective incorporation and distribution of Si-supported Ag particles within the coating. The presence of these characteristics signifies an increase in surface complexity, which may improve interfacial interactions with viruses. The Ag-NaOPZr-Bl coating exhibits the most intricate and compact morphology, distinguished by prominent layered and flake-like structures (Figure 1d). This observation implies a robust integration of the Ag-substituted Zr phosphate phase within the coating, contributing to a highly textured surface architecture. For the Ag-NaOPZr-Bl, the SEM cross-sectional micrograph illustrates the internal microstructure of the sample at a magnification of 2500×. The image reveals a porous architecture characterised by interconnected voids and irregularly shaped pores dispersed throughout the material. This porous network suggests a high surface area and potential for fluid permeability. The scale bar indicates pore sizes in the range of several micrometers, confirming a micro- to mesoporous structure.

The EDS spectrum of the Bl coating validates the dominance of carbon (C) and oxygen (O) as the primary elements, accompanied by lesser amounts of magnesium (Mg), silicon (Si), and sulfur (S) (Figure 1e,f). These elements align with the characteristics of organic–inorganic coating constituents and mineral fillers. The absence of a Ag signal confirms that bioactive additives are not present (Figure 1f). The EDS spectrum for AgSiO_2_-Bl illustrates the elemental makeup of the sample surface, showcasing significant peaks for C, O, Mg, Si, S, and Ag (Figure 1g,h). The pronounced Si peak at approximately 1.75 keV indicates silicon contributions from both the paint fillers and the substrate onto which Ag was applied (Figure 1h). The presence of Ag is corroborated by several peaks around 3.0 keV and 3.2 keV, although these appear at lower intensities, suggesting a minimal Ag content (Figure 1h). Additional signals for C and O suggest the presence of organic or oxide components, while the peaks for Mg and S indicate trace elements. The overall elemental profile points to a complex multi-component material with Ag distributed within a Si-based support. The EDS spectrum of Ag-NaOPZr-Bl displays peaks corresponding to C, O, Mg, Si, and S, along with extra signals attributed to Ag and Zr (Figure 1i,j). The detection of Zr confirms the successful integration of the zirconium phosphate-based phase, while the Ag peaks signify the effective embedding of the bioactive Ag species. These compositional variations affirm the successful functionalization of the coating with Ag-substituted zirconium phosphate.

The quantitative elemental analysis of the tested samples, conducted using SEM-EDS and expressed in weight percentages, is detailed in Table 2. The Bl coating is primarily composed of C (37.7 wt.%) and O (43.6 wt.%), with significant contributions from Mg (4.9 wt.%), Si (8.6 wt.%), and sulfur (3.4 wt.%). At the same time, the levels of Ag, P, and Zr remain below detection limits (refer to Table 2).

The SEM-EDS analysis confirmed the presence of Ag in both Ag-functionalized Bl coatings and provided semi-quantitative information on the elemental composition of the investigated coatings (Table 2). The measured Ag contents differed between Bl coatings, with higher Ag contents detected in Ag-SiO_2_-Bl than in Ag-NaOPZr-Bl. These data confirm the incorporation of Ag into the coatings; however, the EDS measurements were not intended to provide a comprehensive quantitative assessment of the spatial distribution or homogeneity of Ag throughout the coating volume.

Following modification, the Ag-SiO_2_-Bl sample shows an increased C content (43.1 wt.%) and the presence of trace amounts of Na (0.7 wt.%) and Ag (0.04 wt.%), along with slight changes in other elemental components (Table 2 and Table 3). The Ag-NaOPZr-Bl sample reveals a further increase in C concentration (54.3 wt.%), along with detectable quantities of Ag (0.7 wt.%), P (1.6 wt.%), and Zr (3.0 wt.%) (Table 2 and Table 3), while there are corresponding decreases in O, Mg, and Si compared to the earlier samples. These compositional alterations validate the effective incorporation of Ag, Na, P, and Zr elements into the biomaterial post-surface modification.

The values of WCA of Bl, Ag-SiO_2_-Bl, and Ag-NaOPZr-Bl are 57.57 ± 3.93°, 66.47 ± 3.14°, and 59.24 ± 14.53°, respectively (Table 3). As the WCA of all Bl coatings is less than 90°, they can be considered hydrophilic. It can be observed that due to the addition of hydrophobic nAgSiO_2_ in Ag-SiO_2_ 5 wt.%, the WCA was enhanced to 66.47 ± 3.14 compared to the Bl coating. Figure 1k,l depicts the WCA of the Bl coatings.

The colorimetric properties of the Bl coating were evaluated using the CIE Lab colour space and compared with the reference black colour (N0-80-A200), as presented in Table 3. The low L* value confirms the dark appearance of the coating, while the a* and b* coordinates, being close to zero, indicate a nearly neutral black colour with only a slight tendency toward the blue direction (negative b*). The calculated colour differences for Bl coating (ΔE = 1.06), Ag-SiO_2_-Bl (ΔE = 2.02), and Ag-NaOPZr-Bl (ΔE = 1.71) are below the commonly established threshold of ΔE = 3, indicating that the developed coatings closely match the reference black colour and that the difference is barely perceptible under standard viewing conditions. These results confirm the excellent colour quality and suitability of the coating for applications requiring a deep black appearance.

### 2.2. In Vitro Cytotoxicity in Skin and Lung Cellular Models

**Figure 2:**
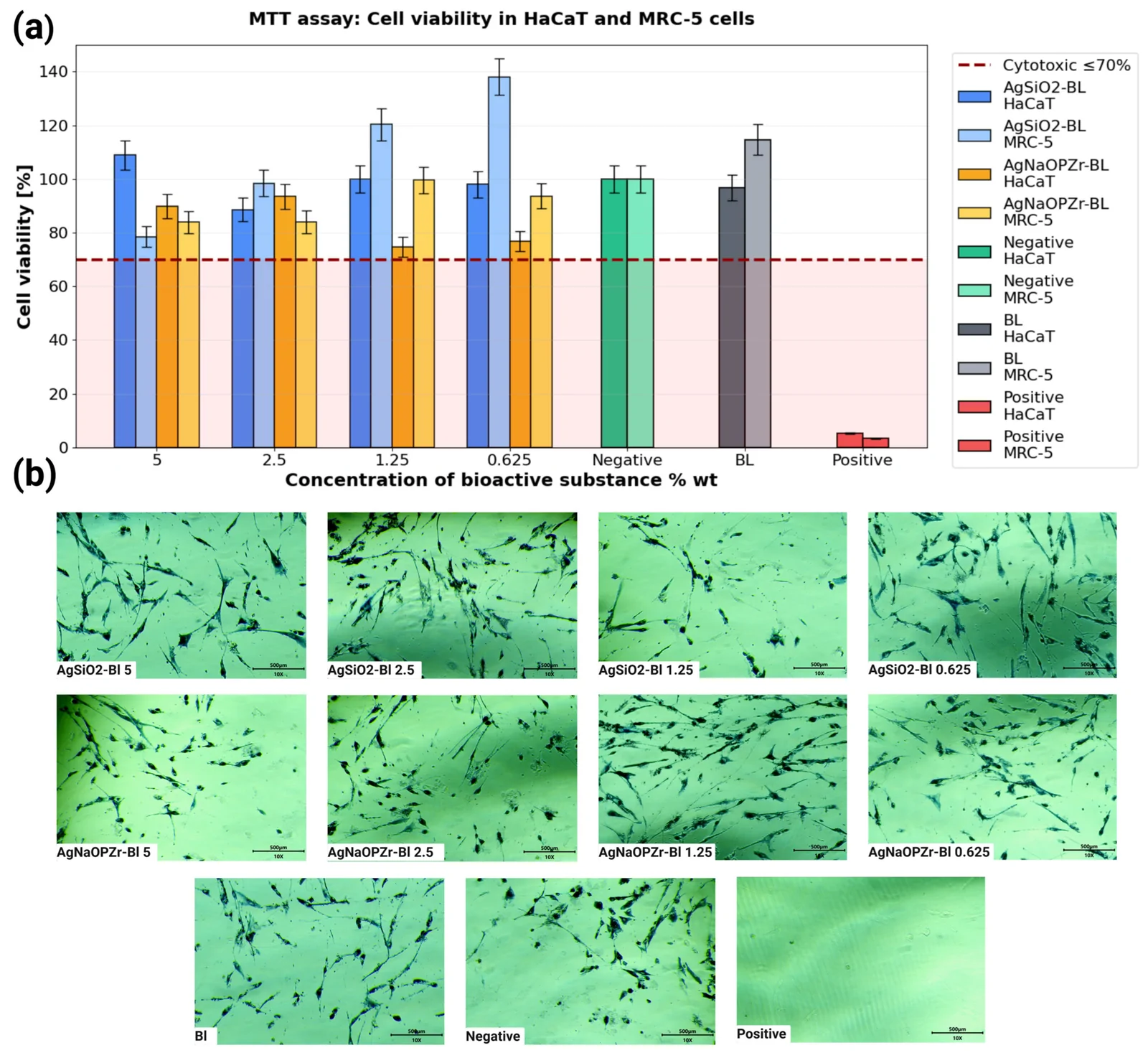
Assessment of in vitro cytotoxicity of Bl coatings using the MTT assay. (**a**) Viability of HaCaT and MRC-5 cells treated with extracts of Ag-SiO_2_-Bl, Ag-NaOPZr-Bl, and Bl coatings. Data represent the mean from three independent experiments (*n* = 3) and three technical replications. The dashed red line indicates the cytotoxicity threshold (70% cell viability).. Bl coatings with cell viability of ≥70% are considered non-cytotoxic. (**b**) Representative images of MRC-5 cells post-MTT staining (2 h) after 24 h exposure to Bl, Ag-SiO_2_-Bl, and Ag-NaOPZr-Bl extracts at indicated concentrations wt.%(). Scale bar = 500 µm. Panels illustrate concentration-dependent formazan production (darker purple = higher metabolic activity). Created in BioRender (2026) https://BioRender.com/cou3qpa.

The results obtained using the MTT assay demonstrated that all Bl coatings tested exhibited non-cytotoxic properties towards HaCaT and MRC-5 cells, with cell viability reaching ≥70% of the control group (negative control) (Figure 2a). For Ag-SiO_2_-Bl, HaCaT cells showed 108.9% viability at 5 wt.%. (highest concentration) while MRC-5 reached 78.5%, with lower dilutions yielding 88.6–138.1% (Figure 2a). Ag-NaOPZr-Bl extracts produced HaCaT viabilities of 74.7–93.5% and MRC-5 values of 83.9–99.6% (Figure 2a). The microscopic assessment of cells stained with MTT was consistent with the spectrophotometric results confirming non-cytotoxic properties of Ag-SiO_2_-Bl, Ag-NaOPZr-Bl, and Bl, alongside negative and positive control specimens through the visible concentration-dependent accumulation of formazan in assessed MRC-5 cells (Figure 2b).

### 2.3. Surface Antiviral Activity

**Figure 3:**
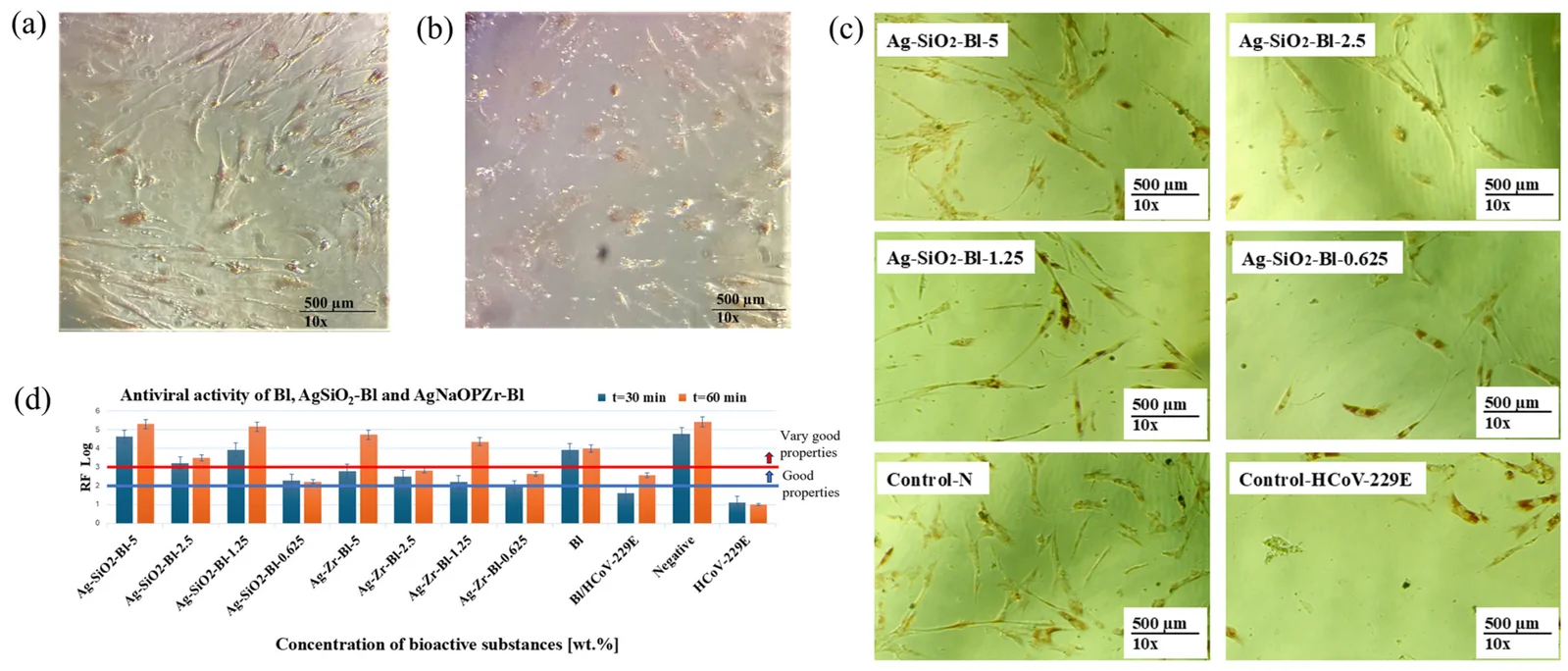
Antiviral activity of Bl coatings. CPE formation on MRC-5 cells assessed using an inverted fluorescence microscope. (**a**) Negative control (MRC-5 cells not inoculated with HCoV-229E). (**b**) Positive control (MRC-5 cells inoculated with HCoV-229E). The findings obtained from the assessment of antiviral activity of Bl coatings after 5–7 days of incubation (T = 35 °C, 5% CO_2_) are presented. (**c**) Representative images obtained using an inverted fluorescence microscope of MRC-5 cells post-NR staining (2.5 h) after 60 min of exposure to Ag-SiO_2_-Bl/HCoV-229E coatings at indicated concentrations wt.%. Scale bar: 500 µm; 10× magnification. (**d**) The graph illustrates the extent of virus titer reduction, calculated as the mean of three independent experiments and three technical replicates for each Bl coating evaluated: Ag-SiO_2_-Bl and Ag-NaOPZr-Bl at concentrations of 0.625, 1.25, 2.5, and 5 wt.%, and Bl coating. The lines depicted on the graph signify the level of antiviral properties of the evaluated coatings. ]; the blue line denotes good antiviral properties (log ≥ 2), while the red line indicates very good antiviral properties (log ≥ 3) of the tested Bl coatings. The bars illustrate the standard error.

Following an incubation period of 5–7 days, 96-well plates were examined for CPE formation utilizing an inverted fluorescence microscope (Figure 3a,b).

The microscopic assessment reveals the absence of CPE resulting from HCoV-229E on cells that were incubated with HCoV-229E lysate that had been treated with Bl, Ag-SiO_2_-Bl, and Ag-NaOPZr-Bl coatings over the t = 60 min across all tested concentrations, in comparison to both positive and negative controls. For treatments at t = 30 min, CPE formation was observed in HCoV-229E lysates treated with Ag-NaOPZr-Bl at ca. 0.625 wt.% and Bl coating, which is analogous to the positive control (HCoV-229E) that was not subjected to any coating treatments. Figure 3c presents examples of microscopic assessment of NR-stained CPE resulting from HCoV-229E infection on MRC-5 cells for antiviral assessment of Ag-SiO_2_-Bl coatings.

The averaged NRU-CPE assay results from replicated tests at t = 30 min assessing the surface antiviral activity of Bl organic coatings against HCoV-229E indicate that Bl achieved the lowest reduction in virus titer levels (log = 1.6) followed by coating Ag-NaOPZr-Bl 0.6 wt.% (log = 1.9) (Table 4) (Figure 3d).

At t = 60 min, Bl, Ag-SiO_2_-Bl, and Ag-NaOPZr-Bl organic coatings with additive loading of bioactive substance ranging from 0.625 to 5 wt.% demonstrated good (log 2.2–2.8) and very good (log 3–5.3) antiviral activity (Table 4) (Figure 3d). The highest reduction level of HCoV-229E was recorded with Ag-SiO2-Bl-treated virus lysates across all tested concentrations (0.625, 1.25, 2.5, and 5 wt.%) of bioactive substances (Table 4) (Figure 3d). The Bl organic coating showed a reduction compared to the negative control level (log ≥ 2) (Table 4) (Figure 3d). The results indicate a decrease in infectious titer from 10^2^ to 10^5^ times (RF ≅ 2–5 log10 TCID_50_/mL) for Bl-, Ag-SiO_2_-Bl-, and Ag-NaOPZr-Bl-treated HCoV-229E lysates (Table 4) (Figure 3d). These values correspond to a 99–99.999% inactivation of the virus titer, confirming the virucidal activity of Bl compounds.

## 3. Discussion

The present study demonstrates that Bl-coatings and those incorporating Ag-SiO_2_ and Ag-NaOPZr as bioactive substances in all tested concentrations (0.625; 1.25; 2.5; and 5% wt.) at t = 60 min exhibit good (log ≥ 2) and very good (log ≥ 3) antiviral activity. The results indicate that the incorporation of Ag-SiO_2_ and Ag-NaOPZr was associated with enhanced antiviral performance of the coatings compared to the unmodified Bl coating. Ag-SiO_2_ demonstrated the most potent antiviral activity, achieving reductions in viral titer of up to 5.3 log, whereas Ag-NaOPZr achieved reductions of up to 4.7 log. Importantly, the direct comparison of the antiviral activity of Ag-SiO_2_-Bl and Ag-NaOPZr-Bl coatings should take into account the differences in their actual Ag content. The Ag content determined by EDS was higher for Ag-SiO_2_-Bl (2.85–3.28 wt.%) than for Ag-NaOPZr-Bl (0.42–1.15 wt.%) across the tested additive concentrations (Table 4). Therefore, the higher antiviral activity observed for Ag-SiO_2_-Bl may be associated, at least in part, with its higher Ag content. Accordingly, the differences in antiviral performance between the two Ag-functionalized coatings should not be interpreted solely as an effect of the type of inorganic additive. The results obtained align with recent research indicating that Ag-based antiviral coatings can significantly diminish the infectivity of HCoV [14,19]. Notably, coatings that are functionalized with Ag-SiO_2_ demonstrated the most potent antiviral efficacy, achieving reductions in viral titer of ≥4 log at specific concentrations while preserving acceptable cytocompatibility with fibroblast cells. The enhanced antiviral activity observed for Ag-SiO_2_-containing coatings may be associated with the physicochemical characteristics of the Ag-SiO_2_ additive and its incorporation into the coating matrix. However, the present study did not directly investigate Ag^+^ release, nanoparticle dispersion at the molecular level, or other mechanisms that could account for the observed antiviral activity [20,21]. Furthermore, it has been previously noted that smaller and well-dispersed AgNPs tend to interact more effectively with viral envelopes, leading to enhanced efficiency in viral inactivation [18]. Crucially, the current findings corroborate earlier studies that suggest Ag-based nanocomposites can maintain favourable biocompatibility despite exhibiting strong antiviral properties [22,23]. Biomaterials containing Ag-SiO_2_ have previously shown good cell viability and adhesion characteristics, which supports their potential use in antiviral biomedical coatings and protective surfaces [24,25]. Nevertheless, some research indicates that the antiviral efficacy of Ag-containing coatings is highly dependent on factors such as nanoparticle size, concentration, and the architecture of the coating [26,27]. Delumeau et al. noted that certain Ag thin-film coatings did not significantly diminish the infectivity of HCoV-229E under particular experimental conditions, while copper coatings exhibited considerably higher antiviral effectiveness. It is noteworthy that the antiviral efficacy in this study demonstrated an inconsistent dose-dependent relationship across the examined formulations. The elevation of nominal Ag-based additive concentrations did not consistently yield a corresponding enhancement in viral infectivity suppression. This finding suggests that the antiviral effectiveness of the coatings is governed by multiple formulation-specific parameters rather than nominal additive concentration in isolation. Variations in measured Ag content, spatial distribution of additives throughout the coating matrix, interfacial properties, and contact duration may collectively account for the observed heterogeneity. Consequently, further optimisation of particle dispersion, ion-release kinetics, and long-term stability of the coatings is essential before their clinical or large-scale environmental applications can be fully realised [28].

The coatings, as waterborne paints, were designed for potential application, particularly for coating polymer substrates such as protective masks and other frequently contacted surfaces, where reducing viral persistence may help limit indirect transmission of respiratory viruses. The persistence of respiratory viruses on solid materials has been widely recognised as an important factor contributing to indirect transmission, particularly in environments with frequent human contact and shared surfaces [29,30,31]. Therefore, the development of antiviral coatings capable of reducing viral stability on surfaces represents a promising approach to complement existing infection-control strategies.

EDS confirmed the successful incorporation of functional additives into the coating. The Bl coating was primarily composed of C and O, with smaller amounts of Mg, Si, and S, suggesting the presence of mineral fillers and pigment components typical of coating formulations. In the Ag-SiO_2_-Bl coating, the presence of Si together with detectable Ag indicates the incorporation of AgNPs supported on SiO_2_. In contrast, the Ag-NaOPZr-Bl coating exhibited additional signals for Ag, P, and Zr, confirming the presence of Ag-substituted zirconium phosphate. These compositional differences are consistent with the designed functionalization strategy and likely contribute to the observed differences in antiviral performance [29,32].

Despite the absence of deliberately incorporated Ag-based antiviral agents within the Bl coating formulation, a quantifiable diminution in viral infectivity was documented (Table 4), suggesting that the coating matrix itself demonstrates inherent antiviral properties under the experimental conditions employed. Notably, the more pronounced reductions observed in the Ag-functionalized coatings indicate an additional contribution from the Ag-based additives to the overall antiviral efficacy. Therefore, the antiviral activity demonstrated by the functionalized coatings should be interpreted as a synergistic effect combining the intrinsic activity of the Bl coating with the contribution derived from Ag-based functionalization. The current experimental methodology, however, does not facilitate the differentiation of the individual molecular mechanisms underlying these observed effects [31].

Additionally, other factors such as intrinsic physicochemical properties of the coating surface, including surface energy, hydrophobicity, and microstructural characteristics, can influence viral adsorption and stability [30]. Such surface-mediated effects have been reported to affect the persistence of enveloped viruses, including HCoV-229E, on solid materials [33]. Adsorption of viral particles to the coating surface or partial destabilisation of the viral envelope may therefore contribute to the reduction in recoverable infectious virus [23,24].

Several studies showed that for biomaterials intended for wearable applications, biocompatibility is an essential consideration [24,34]. In this study, the developed Bl coatings maintain favourable biocompatibility (cell viability > 70%). Similar to other studies reporting non-toxic effects of materials containing Ag or/and SiO_2_ [25], Bl coatings tested for cytotoxicity using HaCaT cells and MRC-5 cells showed no measurable toxic effects under the tested in vitro conditions. These findings suggest that the Bl coatings are compatible with skin and lung contact and may be suitable for applications such as protective masks or functional textiles where contact with skin or proximity to the respiratory system may occur. The cytocompatibility results obtained in the present study should be interpreted within the scope of the applied MTT assay and the two investigated human cellular models, HaCaT keratinocytes and MRC-5 lung fibroblasts. Although these results demonstrate acceptable cell viability under the tested conditions, additional biological assays and cell models could provide a more comprehensive assessment of the biological response to the developed coatings and should be considered in future studies.

WCA is a key parameter governing the interfacial properties of biomaterials, as it strongly influences protein adsorption, the initial attachment of microorganisms, and the subsequent formation of biofilms [35]. Surface wettability determines the organisation of interfacial water molecules, which in turn affects the thermodynamics of biomolecule adsorption and cell adhesion [36]. Generally, hydrophilic surfaces promote the formation of a tightly bound hydration layer that acts as an energetic barrier to nonspecific protein adsorption and microbial attachment [37]. As shown in Table 1, Bl, Ag-SiO_2_-Bl, and Ag-NaOPZr-Bl exhibit hydrophilic behaviour, with WCA values below 90°. Interestingly, the WCA values of all coatings are close to the Berg limit (~65°), which has been identified as an optimum wettability range for minimizing biofouling. Surfaces within this range have been reported to exhibit reduced adsorption of plasma proteins and extracellular polymeric substances, thereby suppressing the early stages of bacterial colonisation and biofilm development [38,39]. This behaviour has been attributed to the presence of a stable hydration layer that limits direct interactions between biological macromolecules and the surface. Although surface wettability alone does not determine antimicrobial and antiviral activity, it plays an important role in reducing the initial adhesion of microorganisms, thereby complementing other antimicrobial mechanisms [40,41]. In the present study, the hydrophilic character of the Ag-containing coatings is expected to act synergistically with the well-established biocidal activity of Ag. While the hydration layer may reduce microbial attachment, released Ag^+^ ions can further inhibit microbial proliferation through membrane disruption, oxidative stress, and interference with intracellular metabolic pathways [42]. Consequently, the combination of favorable surface wettability and silver-mediated antimicrobial activity is likely to enhance the overall resistance of Ag-SiO_2_-Bl and Ag-NaOPZr-Bl coatings to microbial contamination.

## 4. Materials and Methods

### 4.1. Bioactive Substances, Black Coatings Preparation and Characterisation

In the context of bioactive components utilised in Bl coatings, two distinct commercially available antibacterial agents were employed: hydrophobic nano silicon oxide (NanoSilverGuard POWDER 50K-HF) sourced from ITP-System in Dąbrowa Górnicza, Poland, and an Ag-based ion antimicrobial agent (AlphaSan^®^ RC 2000) from Milliken in Spartanburg, NC, USA. The preparation of Bl organic coatings for the evaluation of antiviral activity was conducted following previously established methodologies [19] according to the composition presented in Supplementary Table S1.

#### 4.1.1. Physical and Optical Properties

Bl coatings were characterised for viscosity, density, gloss, and hardness with the method previously reported by Kopyciński et al. 2025 [19].

The surface wettability of the samples (Ø 20 mm) was measured using a contact angle goniometer Model L2004A1 (Ossilia, Leiden, The Netherlands), following a previously reported methodology [43]. Briefly, 10 µL of Milli-Q water was cast on the surface of the Bl coatings using a 25 µL borosilicate syringe (SGE), following the goniometer analysis performed after t = 5 s. The experiment was replicated three times (*n* = 3) for each Bl coating, and the water contact angle (WCA) was calculated as a mean ± standard deviation.

The colour of the Bl coating was determined using an SP62 X-rite portable(X-Rite, Grand Rapids, USA), handheld sphere spectrophotometer with a measuring geometry of d/8 acc. to PN-ISO 7724-2:2003 Paints and Varnishes—Colourimetry—Part 2: Colour measurement [44]. The colour coordinates in CIE Lab colour space were calculated (L*—brightness, a*—color from green to magenta, b*—color from blue to yellow). The color difference ΔE between the paints and the values permitted by the Polish defense standard NO-80-A200:2021 Special camouflage paints—Requirements and test methods was determined under PN-ISO 7724-3:2003 Paints and varnishes—Colorimetry—Part 3: Calculation of color differences [45]. The calculations were made based on the following formula:

$$∆E=\sqrt{{\left({∆L}^{*}\right)}^{2}+{\left({∆a}^{*}\right)}^{2}+{\left({∆b}^{*}\right)}^{2}}$$

#### 4.1.2. Characterisation of Bl Coatings Using SEM and EDS

For SEM analysis, the specimens were intentionally fractured by hand to expose fresh fracture surfaces, which were subsequently attached to aluminum stubs without undergoing any further surface treatment. Observations were performed using a SUPRA-40 (Carl Zeiss AG, Oberkochen, Germany) SEM under high vacuum conditions, with a primary focus on the cross-sections to assess the microstructural features associated with the fracture morphology. The secondary electron imaging mode was utilised to provide intricate topographical contrast.

### 4.2. Maintenance of Cell Lines

The MRC-5 (Cytion, Heidelberg, Germany) was maintained in Minimum Essential Medium (MEM) (Cytivia, VWR, Uppsala, Sweden), which was enriched with 1 g/L D-glucose, 1% non-essential amino acids (Capricorn Scientific GmbH, Ebsdorfergrund, Germany), and 1 mM sodium pyruvate (Capricorn Scientific), and/or EMEM (Cytion). The HaCaT (Medical University of Warsaw, Warsaw, Poland) cell line was cultivated in Dulbecco’s Modified Eagle Medium (DMEM) high glucose (Capricorn Scientific). Both cell lines were supplemented with 10% heat-inactivated fetal bovine serum (FBS) (EurX^®^, EURx Sp. z o.o., Gdańsk, Poland), penicillin (100 U/mL), and streptomycin (100 μg/mL) (Sigma-Aldrich, St. Louis, MO, USA). The cell lines were grown in six-well plates (Sarstedt, Nümbrecht, Germany), 96-well microplates (Sarstedt), and 25–75 cm^2^ adherent culture flasks (Sarstedt) at a temperature of 37 °C with 5% CO_2_, before infection with HCoV-229E for either multiplication or antiviral testing of Bl organic coatings. Daily examinations of the cells were performed using Visiscope^®^ IT407 FL (VWR International, LLC, Radnor, PA, USA), an inverted trinocular fluorescence microscope (VWR).

### 4.3. Reference HCoV-229E Strain and Its Replication Within a Cell Line

For virus stock multiplication, the reference strain of HCoV-229E (ATCC-VR-740) (LGC Standards, Teddington, UK) was cultivated on adherent host cells MRC-5 (Cytion) by incubating at temperature 35 °C with 5% CO_2_ for a duration of 1–2 h to facilitate virus adsorption into the cells. The multiplicity of infection (MOI) was determined to be 1. The adsorption process of HCoV-229E was halted by the addition of an infection medium that consisted of [EMEM (Cytion)/MRC-5 (Cytion) combined with 2% FBS (EurX^®^)]. Following the virus adsorption phase, 25 cm^2^ bottles were incubated at 35 °C and 5% CO_2_ for a period of 5–7 days. The CPE resulting from the viral infection of the cell lines was monitored using a reverse-phase contrast fluorescence microscope AE 2000 (Motic, Wetzlar, Germany) on consecutive days of infection. After the incubation period of 5–7 days, the supernatant containing the viruses was collected, subjected to centrifugation (10,000× *g*, at t = 4 °C) using centrifuge 5425 (Eppendorf, Hamburg, Germany), and filtered through a sterile PES 25 mm, 0.22 µm syringe filter (VWR). The viral titers were quantified as 50% tissue culture infective dose (TCID_50_)/mL (Spearman-Karber) [46] before conducting antiviral tests on the Bl organic coating discs. The TCID_50_/mL of the viral supernatants obtained was 2.5 × 10^5^. All work with HCoV-229E has been carried out at a biosafety level 2 laboratory.

### 4.4. Testing the Surface Antiviral Activity of Bl Organic Coatings

The antiviral activity of Bl organic coatings has been assessed under the adopted ISO 21702:2019 EN methodology for ‘Measurement of antiviral activity on plastics and other non-porous surfaces’ with modifications as reported previously [14,47]. Before testing antiviral activity, Bl organic coating discs were disinfected using a UV lamp at 254 nm under a biosafety cabinet for 1 h. The antiviral assays were conducted by introducing previously acquired virus supernatant, approximately TCID_50_/mL 2.5 × 10^5^ (900 µL), onto each surface of the Bl organic coating discs under examination, allowing it to remain for 30 and 60 min at a temperature of 20–22 °C. The culture medium was discarded from 96-well plates (prepared 48 h before testing), and 100 µL of HCoV-229E supernatant that had been in contact with the Bl organic coating discs was added in triplicate for each concentration (0%, 0.625%, 1.25%, 2.5% and 5%) after 30–60 min. Subsequently, 100 µL of serially diluted 1/10 (N, 10^−1^, 10^−2^, 10^−3^, 10^−4^) HCoV-229E stock in infection medium was introduced to each well as a positive control, while 100 µL of virus-free infection medium served as a negative control. The plate was then incubated in a cell culture incubator (humidified, 5% CO_2_, T = 35 °C) for 1–2 h to facilitate virus adsorption. Following this incubation period, 100 µL of infection medium was added to each well of the 96-well plate, resulting in a total volume of 200 µL. The plates were subsequently incubated for a duration of 5–7 days. Daily examinations of the cells post-infection were performed using a Visiscope^®^ IT407 FL inverted trinocular fluorescence microscope (VWR), documenting any alterations in cell morphology and the development of CPE. After 5–7 days of incubation, plates were tested with the method previously reported for testing antiviral compounds’ activity against HCoV-229E, with several modifications [48]. The NR solution, which contained NR (Warchem, Zakręt, Poland) at approximately 150 µg/mL, EMEM (Cytion), 2% FBS (EurX^®^), was incubated for 12 h at 37 °C, followed by centrifugation (4000× *g*, 5 min, T = 20–30 °C) and filtration through a sterile PES 25 mm, 0.1 µm syringe filter (Thermo Fisher, Waltham, MA, USA) to eliminate any NR precipitates formed. The virus infection medium was then discarded from the incubated 96-well plates, and the cells were washed by adding 200 µL of phosphate-buffered saline (PBS), pH = 7.2 (EurX^®^), containing calcium and magnesium to each well. After the washing step, 100 µL of the NR solution prepared before was introduced to each well of the plate. The plate was subsequently incubated in a cell culture incubator at 37 °C (5% CO_2_) for 2.5 h. After this time, the NR solution was removed, and the cells were washed by adding 200 µL of PBS (EurX^®^) with calcium and magnesium to each well. After washing, 100 µL of extraction solution [70% ethanol (Warchem), 10% glacial acetic acid (Warchem), and 20% distilled sterile water] was added to each well for 15 min of extraction. The plates were analysed for optical signal intensity using a smartphone Spotxel^®^ Microplate Reader 2.2.2 (SICASYS Software GmbH, Germersheim, Germany) [49]. Analysis of the obtained antiviral results was performed for three technical replications of three independent trials. Normalisation was performed by comparing the optical signal values obtained with the smartphone Spotxel^®^ Microplate Reader 2.2.2. (SICASYS Software GmbH) [49] of the test samples to the control samples. The ISO 21702 virucidal efficacy testing includes three controls: a cytotoxicity control that assesses any potential harm to host cells; an untreated sample control to compare the antiviral activity with the treated sample; and a titration control to ensure that the virus stock concentration is sufficient to determine infectivity and measure the highest viral recovery rate from the sample. These controls provide key benchmarks for accurate and reliable results in virucidal testing [47]. For antiviral tests: negative control (MRC-5 lung fibroblast cells with infection medium), Bl (coating paint 0% wt. of bioactive compounds extract and HCoV-229E), positive control (cells with medium and HCoV-229E). Calibration curves of HCoV-229E lysate dilutions as positive controls were determined for each test. Reduction factor (RF) was calculated as the logarithmic titer in the virus control minus the logarithmic titer of the treated sample with virus (RF = Δ log10 TCID_50_/mL). A log reduction of 2 or higher must be obtained in the viral particles for the material to pass the ISO 21 702 virucidal efficacy testing [47]. The experimental results are presented as mean ± standard deviation (SD). Graphical data are presented as mean values with corresponding standard deviation bars.

### 4.5. Cytotoxcity

The cytotoxicity of Bl coatings was evaluated through the use of 3-(4,5-dimethylthiazol-2-yl)-2,5-diphenyltetrazolium bromide (MTT) (Chemat, Gdańsk, Poland) assay in accordance with ISO 10993-5: Biological evaluation of medical devices-Part 5: Tests for in vitro cytotoxicity with several modifications [50]. This assessment included modifications that utilized MRC-5 (Cytion) and HaCaT (Medical University of Warsaw) cell lines. MRC-5 (Cytion) and HaCaT (Medical University of Warsaw) cells were seeded at a density of 1×10^4^ cells per well in 96-well plates and incubated for a duration of 24–48 h at 37 °C with 5% CO_2_ to facilitate cell attachment. Extracts were prepared by incubating Bl coating discs in complete culture media containing EMEM (Cytion); 10% FBS (EurX^®^); 1% penicillin/streptomycin (Capricorn Scientific) for MRC-5; DMEM High Glucose (Capricorn Scientific); 10% FBS (EurX^®^); 1% penicillin/streptomycin (Capricorn Scientific) for HaCaT (Medical University of Warsaw) for 24 h. Subsequently, cells were treated with 100 µL of previously prepared extracts (5, 2.5, 1.25, 0.625 wt.%), in addition to negative controls (complete medium) and a positive control containing 100% dimethyl sulfoxide (DMSO) (Warchem), for 24 h at 37 °C with 5% CO_2_. After the incubation period, the media were removed, and the cells were treated with 50 µL of MTT solution (prepared as 5 mg/mL MTT (Chemat) in PBS (EurX^®^), diluted in culture medium without supplements to 1 mg/mL) for 2 h at 37 °C with 5% CO_2_. The MTT (Chemat) solution was carefully aspirated to retain the formazan crystals, followed by the addition of 100 µL of DMSO (Warchem) per well to utilise the crystals. Absorbance was recorded at 570 nm using a Varioskan LUX Multimode Microplate Reader (Thermo Fisher Scientific, Waltham, MA, USA). The absorbance values were utilised, and cell viability was calculated as a percentage relative to the negative controls; materials that exhibited ≥70% viability at the highest extract concentration were classified as non-cytotoxic. The experimental results are presented as mean ± standard deviation (SD). Graphical data are presented as mean values with corresponding standard deviation bars.

## 5. Conclusions

In summary, the results demonstrate that integrating Ag-based bioactive substances into Bl coatings represents a promising strategy for the development of antiviral coatings. Such coatings combine effective viral inactivation with favourable biocompatibility, making them attractive candidates for next-generation protective materials designed to reduce viral persistence on personal protective equipment and high-contact surfaces. A notable limitation of this study pertains to the absence of direct examination of the particular mechanisms responsible for the demonstrated antiviral efficacy. Specifically, supplementary research focusing on the quantification of Ag^+^ liberation and the production of ROS would be necessary to elucidate their respective roles in mediating the antiviral properties of the Bl coatings.

A limitation of the present study is that the specific mechanisms underlying the observed antiviral activity were not directly investigated. In particular, further studies involving the quantification of Ag^+^ release and ROS generation would be required to determine their potential contribution to the antiviral activity of the developed coatings. In addition, coating durability and the persistence of antiviral activity over extended periods were not evaluated. Therefore, the present results do not allow conclusions to be drawn regarding long-term coating stability or the retention of antiviral activity following prolonged storage, repeated handling, or other environmental and use-related conditions. These aspects should be addressed in future studies.

In the present study, the antiviral activity was evaluated under standardized experimental conditions, while a systematic correlation between surface roughness/morphology and antiviral performance was not specifically investigated. Therefore, although surface topography may contribute to the observed antiviral effect, further studies involving controlled variation in surface roughness and quantitative morphological characterization would be required to establish a direct structure–activity relationship.

## Figures and Tables

**Table 1:** Characteristics of physicochemical properties of Bl, Ag-SiO_2_-Bl-5 wt.%., and Ag-NaOPZr-Bl-5 wt.% coatings.

Property	Coating Id		
	Bl ^1^	Ag-SiO_2_-Bl ^2^	Ag-NaOPZr-Bl ^3^
**Viscosity [mPa·s]**	1818 (A/5/50)	12.220 (A/5/10)	1948 (A/5/50)
**Density** **[g/cm^3^]**	1.23	1.22	1.23
**Non-volatile content** **[%]**	50	50	49
**Gloss** **[20°/60°/85°]**	0.5/5.6/11.3	0.3/3.4/9.2	0.4/5.2/24.4
**Hardness** **[s]**	167 ± 2	172 ± 1	162 ± 1
**Relative hardness** **[% of 430 s]**	0.39	0.40	0.38

^1^ Bl coating with 0 wt.% bioactive additive; ^2^ Bl coating containing 5 wt.% Ag-SiO_2_; ^3^ Bl coating containing 5 wt.% Ag-NaOPZr.

**Table 2:** Mean elemental weight percentages in Bl, Ag-SiO_2_-Bl (5 wt.%), and Ag-NaOPZr-Bl (wt.%) coatings, as determined by SEM–EDS analysis.

Coating Id	Element’s Weight [%] ^4^							
	C	O	Na	Mg	Si	S	P	Zr Ag
Bl ^1^	37.7	43.6	0.0	4.9	8.6	3.4	0.0	0.0 0.0
Ag-SiO_2_-Bl ^2^	43.1	41.7	0.7	3.8	8.2	3.1	0.0	0.0 0.04
Ag-NaOPZr-Bl ^3^	54.3	37.2	0.6	2.1	5.5	4.2	1.6	3.0 0.65

^1^ Bl coating with 0 wt.% bioactive additive; ^2^ Bl coating containing 5 wt.% Ag-SiO_2_; ^3^ Bl coating containing 5 wt.% Ag-NaOPZr; ^4^ Elemental weight percentages in Bl coatings determined by SEM–EDS analysis.

**Table 3:** Selected characteristics of Bl coatings: Ag content, particle size, wettability assessed by water contact angle (WCA), colour measurements, and colour matching to the reference standard (NO-80-A200) of Bl, Ag-SiO_2_-Bl (5 wt.% ), and Ag-NaOPZr-Bl (5 wt.%) coatings.

Coating Id	Content of Ag in Bioactive Additive [%] ^1^	Particle Size [nm] ^2^	WCA (A°) ± SD ^3^	CIE Lab Colour acc. PN-ISO 7724-2 ^4^			Reference Color acc.NO-80-A200 ^5^		
				L*	a**	b***	L*	a**	b***
Bl ^6^	0.00	n/a	57.57 ± 3.93°	24.54	0.04	0.61	23.67	0.00	0.00
Ag-SiO_2_-Bl ^7^	5.00	5–15	66.47 ± 3.14°	25.55	−0.12	0.74	n/a	n/a	n/a
Ag-NaOPZr-Bl ^8^	10.0	1300	59.24 ± 14.53°	24.99	0.08	−0.55	n/a	n/a	n/a

^1^ Percentage content of Ag in bioactive compound used for preparation of Bl coatings; ^2^ Particle size of loading additive used for preparation of Ag-SiO_2_-Bl and Ag-NaOPZr-Bl accessed from certificate of analysis; ^3^ Wettability assessed by measuring the water contact angle (WCA). Data are presented as mean ± standard deviation (SD). ^4^ The color coordinates in CIE Lab color space were calculated (L*—brightness, a**—color from green to magenta, b***—color from blue to yellow); ^5^ Reference color acc.NO-80-A20 were calculated (L*—brightness, a**—color from green to magenta, b***—color from blue to yellow). ^6^ Bl coating with 0 wt.% bioactive additive; ^7^ Bl coating containing 5 wt.% Ag-SiO_2_; ^8^ Bl coating containing 5 wt.% Ag-NaOPZr.

**Table 4:** Results obtained from the assessment of the antiviral activity of Bl, Ag-SiO_2_-Bl, and Ag-NaOPZr-Bl organic coatings, exposed to HCoV-229E under controlled contact conditions for 30 and 60 min. For assay control, HCoV-229E was used. The NRU–CPE assay was performed in triplicate (*n* = 3) and repeated in three independent experiments.

Tested Coating Id */ Assay Control Id **	Additive Loading [wt%] ^1^	RF [log] t = 30 min	ISO Pass 30 min	RF [log] t = 60 min	ISO Pass 60 min
AgSiO2-Bl *^2^	5.0	4.6	Yes	5.3	Yes
AgSiO2-Bl *^2^	2.5	3.2	Yes	3.5	Yes
AgSiO2-Bl *^2^	1.25	3.9	Yes	5.1	Yes
AgSiO2-Bl *^2^	0.625	2.3	Yes	2.2	Yes
Ag-NaOPZr-Bl *^3^	5.0	2.8	Yes	4.7	Yes
Ag-NaOPZr-Bl *^3^	2.5	2.5	Yes	2.8	Yes
Ag-NaOPZr-Bl *^3^	1.25	2.2	Yes	4.3	Yes
Ag-NaOPZr-Bl *^3^	0.625	1.9	No	2.6	Yes
Bl/HCoV-229E *^4^	0.0	1.6	No	2.5	Yes
Negative **^6^	0.0	4.7	n/a ^8^	5.4	n/a ^8^
HCoV-229E **^5^	0.0	1.1	n/a ^8^	1.0	n/a ^8^
Bl **^7^	0.0	3.9	n/a ^8^	4.0	n/a ^8^

^1^ Concentration of additive loading of bioactive substance used in prepared Bl coatings expressed in weight percentage [wt.%]; ^2^ Bl coatings containing Ag-SiO_2_ exposed to HCoV-229E before performing NRU-CPE assay; ^3^ Bl coatings containing Ag-NaOPZr exposed to HCoV-229E before performing NRU-CPE assay; ^4^ Bl coating (no additive loading of bioactive substance) exposed to HCoV-229E before performing NRU-CPE assay; ^5^ HCoV-229E used as a positive assay control; ^6^ Additional control including MRC-5 cells and medium without HCoV-229E; ^7^ Bl coating (no additive loading of bioactive substance) without HCoV-229E; ^8^ Not applicable. * Tested coating; ** Control Assay.

## Data Availability

The raw data are available from the corresponding author and the authors upon request.

## Supplementary Materials

The following supporting information can be downloaded at: https://www.mdpi.com/article/10.3390/ijms27219399/s1.
