# Supplementary material for: Bioactive Antiviral Black Organic Coatings Functionalized with Silver–Silica and Silver–Zirconium for Cytocompatible Biomaterial Surfaces Against Human Coronavirus
Source: Int J Mol Sci. 2026 Oct 22;27(21):9399. doi: 10.3390/ijms27219399 (PMC13762975; doi:10.3390/ijms27219399)
Supplement: Supplementary file 1 [file ijms-27-09399-s001.zip › ijms-4625387-supplementary.pdf]

Supplementary Table S1. Formulas of developed Bl organic coating.

Component of paint	Quantity, kg/100 kg
	Bl
Setaqua® 6754, Allnex	46.0
Dipropyleneglycol DME, Clariant	11.0
Tafigel® Pur 60, Münzing Chemie	0.3
Pigments	2.5
Disperbyk-192, Byk-Chemie	0.5
Byk-028, Byk-Chemie	1.0
Byk-346, Byk-Chemie	1.0
Aerosil® R 972, Evonik	1.0
Sachtleben Micro, Sachtleben Chemie	11.0
Finntalc M15, Elementis	9.0
Dimethylethanolamine 99%, Thermo Fisher Scientific, 10% (v/v) in demi water	0.5
Demi water	16.2
